# Impact of Scotland's Smoke-Free Legislation on Pregnancy Complications: Retrospective Cohort Study

**DOI:** 10.1371/journal.pmed.1001175

**Published:** 2012-03-06

**Authors:** Daniel F. Mackay, Scott M. Nelson, Sally J. Haw, Jill P. Pell

**Affiliations:** 1 Centre for Population and Health Sciences, University of Glasgow, Glasgow, United Kingdom; 2 Scottish Collaboration on Public Health Research Policy, Western General Hospital, Edinburgh, United Kingdom; Simon Fraser University, Canada

## Abstract

**Background:**

Both active smoking and environmental tobacco smoke exposure are associated with pregnancy complications. In March 2006, Scotland implemented legislation prohibiting smoking in all wholly or partially enclosed public spaces. The aim of this study was to determine the impact of this legislation on preterm delivery and small for gestational age.

**Methods and Findings:**

We conducted logistic regression analyses using national administrative pregnancy data covering the whole of Scotland. Of the two breakpoints tested, 1 January 2006 produced a better fit than the date when the legislation came into force (26 March 2006), suggesting an anticipatory effect. Among the 716,941 eligible women who conceived between August 1995 and February 2009 and subsequently delivered a live-born, singleton infant between 24 and 44 wk gestation, the prevalence of current smoking fell from 25.4% before legislation to 18.8% after legislation (*p*<0.001). Three months prior to the legislation, there were significant decreases in small for gestational age (−4.52%, 95% CI −8.28, −0.60, *p* = 0.024), overall preterm delivery (−11.72%, 95% CI −15.87, −7.35, *p*<0.001), and spontaneous preterm labour (−11.35%, 95% CI −17.20, −5.09, *p* = 0.001). In sub-group analyses, significant reductions were observed among both current and never smokers.

**Conclusions:**

Reductions were observed in the risk of preterm delivery and small for gestational age 3 mo prior to the introduction of legislation, although the former reversed partially following the legislation. There is growing evidence of the potential for tobacco control legislation to have a positive impact on health.

*Please see later in the article for the Editors' Summary*

## Abstract 2

An analysis of pregnancy data for the whole of Scotland demonstrates a reduction in small-for-gestational-age births and preterm delivery since the introduction of legislation banning smoking in enclosed public spaces.

## Editors' Summary

**Background:**

The risks of smoking during pregnancy, both on mother and fetus, are well established: women who smoke during pregnancy are more likely to have a miscarriage. Smoking can cause placental problems, such as placental abruption, which can result in heavy bleeding during pregnancy, which is dangerous for both mother and baby. Other dangers of smoking during pregnancy include the baby being born too early (premature birth), the baby being below average weight (small for gestational age), birth defects, and infant death. Because of the serious damage to health caused by smoking, in 2005, under the auspices of the World Health Organization, countries adopted the Framework Convention on Tobacco Control to protect present and future generations from the devastating health, social, environmental, and economic consequences of tobacco consumption and exposure to tobacco smoke. Article 8 of the treaty obliges member states who have ratified the treaty—168 so far—to protect all people from exposure to tobacco smoke in indoor workplaces, public transport, and indoor public places. As a result, many countries around the world have banned smoking in public places.

**Why Was This Study Done?:**

Scotland was the first country in the United Kingdom to ban smoking in public places, which was implemented as part of the Smoking, Health and Social Care (Scotland) Bill on 26 March 2006. Previous studies have shown that the introduction of the legislation led directly to a reduction in smoking and also a reduction in environmental tobacco smoke exposure in adults and children. Furthermore, the Scottish legislation has been accompanied by significant reductions in both cardiovascular and respiratory disease. Because of the known risks of smoking during pregnancy, the researchers wanted to investigate whether the change in policy on smoking in public places had positive benefits on the health of mothers and babies. They evaluated this by measuring the rates of preterm delivery and small for gestational age before and after the Scottish legislation went into effect.

**What Did the Researchers Do and Find?:**

The researchers collected information on preterm delivery and small for gestational age in all single babies born live at 22–44 weeks gestation between 1 January 1996 and 31 December 2009 by using the Scottish Morbidity Record (SMR2), which collects relevant information on all women discharged from Scottish maternity hospitals, including maternal and infant characteristics and pregnancy complications. The researchers categorized preterm delivery into mild, moderate, and extreme depending on how much before 37 weeks the baby was born. They defined small for gestational age as the smallest 10% (below the 10th centile) for sex-specific birth weight at delivery, and very small for gestational age as the smallest 3% (below the 3rd centile), for all deliveries in Scotland over the study period. As some people may have stopped smoking in anticipation of the smoking ban, in their statistical model, the researchers included two possible breakpoints for the effect of the legislation—the actual date of implementation and 1 January 2006.

The researchers found that of the 716,968 pregnancies (the number eligible for inclusion in the study), 99.9% of women had their smoking status recorded, and among these 23.9% were current smokers, 57.6% never smokers, and 8.7% former smokers. However, following implementation of the legislation the researchers noted that there was a significant reduction in current smokers to 18.8%. In their statistical model, the researchers found that following 1 January 2006, there was a significant drop in overall preterm deliveries, which remained after adjustment for potential confounding factors. Likewise, there was a significant decrease in the number of infants born small, and very small, for gestational age after 1 January 2006. Furthermore, the researchers found that these significant reductions occurred in both mothers who smoked and those who had never smoked.

**What Do These Findings Mean?:**

These findings suggest that the introduction of national, comprehensive smoke-free legislation in Scotland was associated with significant reductions in preterm delivery and babies being born small for gestational age. These findings are plausible and add to the growing evidence of the wide-ranging health benefits of smoke-free legislation, and support the adoption of such legislation in other countries that have yet to implement smoking bans.

**Additional Information:**

Please access these websites via the online version of this summary at http://dx.doi.org/10.1371/journal.pmed.1001175.

- More information is available on the World Health Organization's Framework Convention for Tobacco Control
- More information on the Smoking, Health and Social Care (Scotland) Bill is available
- The US Centers for Disease Control and Prevention has more information about the risks of smoking in pregnancy, as does the UK National Health Service's smokefree web page
- NHS Health Scotland has a website that summarises all the studies to date evaluating the Scottish smoke-free legislation

## Introduction

There is substantial evidence that both active smoking during pregnancy and maternal exposure to environmental tobacco smoke (ETS) increase the risk of pregnancy complications [1]–[7]. In Scotland, the Smoking, Health and Social Care (Scotland) Bill prohibited smoking in all enclosed public places and workplaces from 26 March 2006. The legislation has been extremely successful in its primary aim of reducing exposure to ETS in public places, with an 86% reduction in air PM_2.5_ concentrations occurring within 2 wk [8]. Initial concerns that the legislation would merely displace smoking into homes have not been realized. Conversely, the legislation has resulted in greater adoption of voluntary restrictions on smoking in the home [9], and therefore decreased overall exposure among both adults [10] and children [9]. In addition to reduced ETS exposure, an increase in quit attempts among current smokers occurred 3 mo prior to introduction of the legislation [11], and there was a reduction in the amount smoked by those who continued to smoke [10]. The legislation has been accompanied by significant reductions at a population level in both cardiovascular [12] and respiratory disease [13],[14]. One previous study has examined the effect of smoke-free legislation on pregnancy complications. The study, conducted in Ireland, suggested a reduction in preterm deliveries and an increase in low birth weight, but had a number of limitations [15]. The Scottish Morbidity Record (SMR2) provides an invaluable resource for determining whether the risk of pregnancy complications, among current and never smokers, has changed following implementation of the Scottish smoke-free legislation.

## Methods

### Data Source

The SMR2 collects information on all women discharged from Scottish maternity hospitals, including maternal and infant characteristics, obstetric history, clinical management, and pregnancy complications. The SMR2 is subjected to regular quality assurance checks. The most recent, performed in 2010, compared a 4.4% sample of SMR2 returns (*n* = 2,531) with case records and demonstrated high quality for all the data fields used in our study: in particular, infant sex was 100% complete and accurate, birth weight 99%, and gestation at time of delivery 92% [16].

### Inclusion Criteria and Definitions

We obtained SMR2 data on all infants delivered in Scotland between 1 January 1996 and 31 December 2009, the latter equating to the most recent data available at the time of analysis. Our analyses were restricted to singleton, live-born infants delivered at 24–44 wk of gestation. Both nulliparous and multiparous women were included. Date of conception was derived by subtracting the gestation at delivery from the date at delivery and then adding 2 wk to adjust for the normal menstrual cycle. Early deliveries were systematically underrepresented among the earliest conception dates, and late deliveries among the latest conception dates. Therefore, we restricted our analyses to conceptions occurring between 1 August 1995 and 10 February 2009, as this was the maximum range of conception dates across which all deliveries occurring between 24 and 44 wk of gestation would be included. Smoking status was based on self-classification at booking and comprised current, never, and former smokers. Postcode of residence is recorded on the SMR2 record and is used to allocate individuals to a socioeconomic quintile of the general population using the 2004 Scottish Index of Multiple Deprivation (http://www.scotland.gov.uk/Publications/2005/01/20458/49127). The index is derived from 31 area markers of deprivation relating to health, education, housing, current income, employment access, and crime that are applied to each postcode data zone. There are 6,505 data zones in Scotland, with a mean population of 750 individuals each.

The primary outcomes for this study were preterm delivery and small for gestational age. Secondary outcomes included low birth weight; spontaneous preterm labour; mild, moderate, and extreme preterm delivery; and very small for gestational age. Preterm delivery was defined as delivery at less than 37 wk gestation, and was categorised into mild (≥34 and <37 wk gestation), moderate (≥32 and <34 wk gestation), and extreme (<32 wk gestation). In the SMR2 record, gestational age at birth is defined as completed weeks of gestation on the basis of the estimated date of delivery recorded in each woman's clinical record. Gestational age has been confirmed by ultrasound in the first half of pregnancy in more than 95% of women in the United Kingdom since the early 1990s. Small for gestational age was defined as below the 10th centile for gestation- and sex-specific birth weight at delivery, and very small for gestational age as below the 3rd centile, with centiles derived from all deliveries in Scotland over the period studied. Low birth weight was defined as <2,500 g.

### Statistical Analyses

Separate logistic regression models were created for each pregnancy outcome. The models allowed for an underlying trend in the odds for each pregnancy outcome throughout the whole study period, and for a possible step and slope change in the odds due to implementation of the smoke-free legislation. We examined two possible breakpoints for the effect of the legislation: the actual date of implementation (26 March 2006) and 1 January 2006. The latter date allowed for the possibility of anticipatory changes in smoking behaviour and was chosen to coincide with the peak in smoking quit attempts observed in a previous study [11]. We compared the two breakpoints using Akaike information criterion statistics, with the lowest Akaike information criterion statistic indicating the model with the best fit. In the multivariable models we adjusted for maternal age, sex of the infant, deprivation quintile, week of conception, number of previous spontaneous abortions, number of previous therapeutic abortions, and parity. Pre-eclampsia, a possible negative mediator, was then added to the model as an additional covariate. The models were run initially for all eligible pregnancies, including current, never, and former smokers as well as women with missing information on smoking status. We then performed separate sub-group analyses for current smokers and never smokers. The beta coefficients derived from the logistic regression models for the step change at the breakpoint and the post-breakpoint change in slope were converted into the percentage change in odds using the formula 100×(exp(β)−1). In order to test the robustness of our results, we imputed smoking status for women for whom this was missing using multiple imputation by chained equations and then re-ran all the analyses. The chained equations used the same variables as the full models. Five imputed datasets were created, and a sensitivity analysis, comparing complete cases with the imputed datasets, was conducted. All analyses were undertaken using Stata v11.2 (StataCorp).

## Results

Between 1 January 2006 and 31 December 2009, there were 756,795 deliveries. We excluded 282 (0.04%) because of missing information on gestation at delivery and a further 15,211 (2.0%) because they did not satisfy the inclusion criteria (singleton, live-born infant delivered at 24–44 wk gestation). 24,334 deliveries were excluded because the conception date was earlier than 1 August 1995 or later than 10 February 2009. Among the remaining 716,968 pregnancies, smoking status at booking was recorded for 716,941 (99.9%) women. Among these, 171,454 (23.9%) were current smokers, 412,800 (57.6%) never smokers, and 62,227 (8.7%) former smokers. Following implementation of the legislation there was a reduction in current smokers (from 25.4% to 18.8%, *p*<0.001) and an increase in never smokers (from 57.3% to 58.4%, *p*<0.001). Overall, 42,715 (6.0%) infants were born preterm. Of these, 30,659 (71.8%) were mild preterm, 5,614 (13.1%) moderate preterm, and 6,442 (15.1%) extreme preterm. Of the 42,715 preterm deliveries, 801 (1.9%) had missing information on mode of delivery. Among the remaining 41,914 mothers, 21,538 (51.4%) were delivered electively and 20,971 (50.0%) went into spontaneous labour.

**Figure 1:**
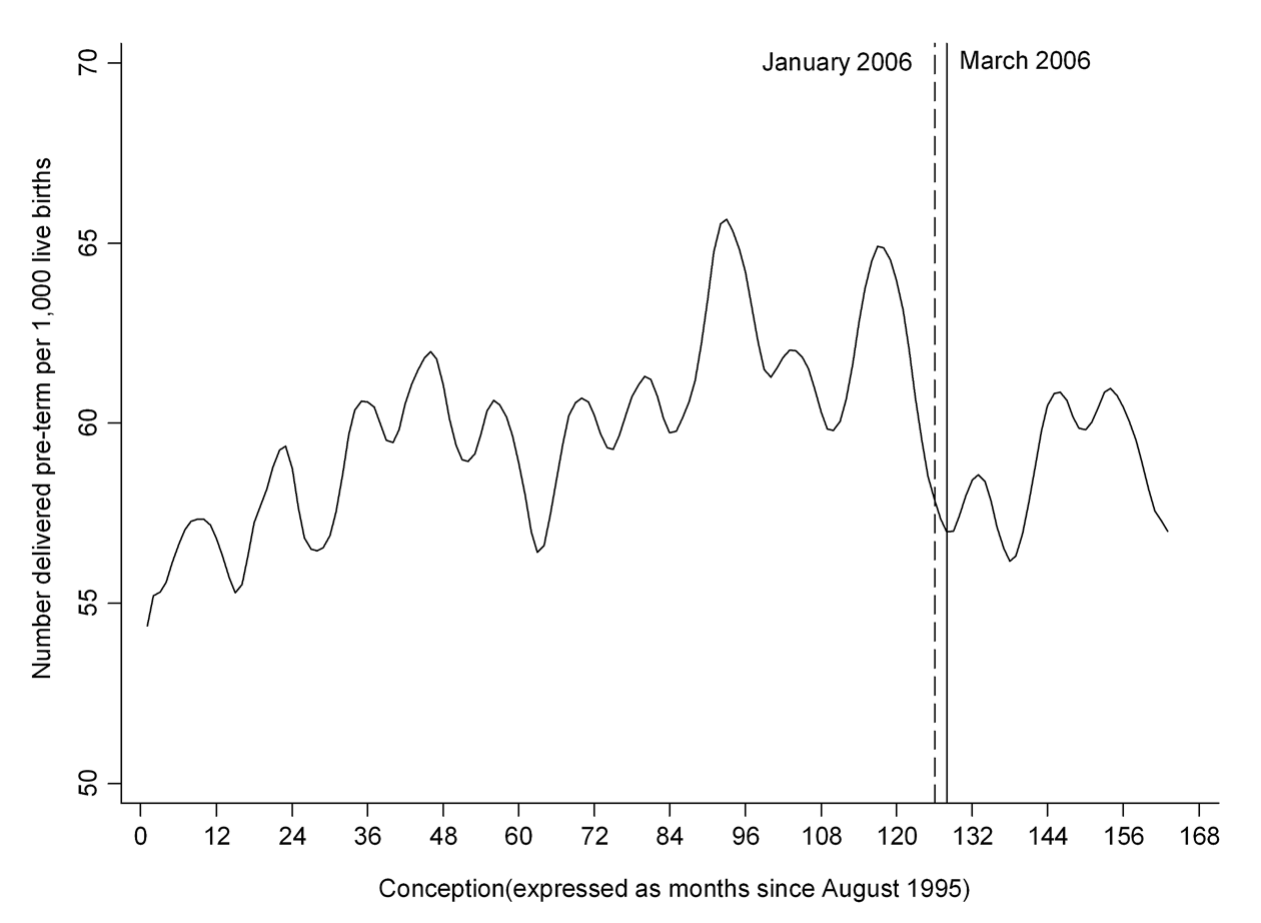
Time trend in the number of infants delivered preterm per 1,000 live births. Time trend smoothed using the Stata loess smoother with bandwidth = 0.1.

The Akaike information criterion statistics suggested that using 1 January 2006 as the breakpoint produced a marginally superior model fit than using 26 March 2006. Therefore, the results of the former model are reported. Figure 1 depicts the crude proportion of infants born preterm by month of conception. From the figure, it appears that preterm deliveries were increasing prior to the legislation, followed by a sharp decline in the months immediately prior to legislation, although this appeared to have been partially reversed around 2 y later. From the model, there was an 11.07% (95% CI 6.79, 15.15, *p*<0.001) decrease in overall preterm deliveries and a 10.26% (95% CI 4.04, 16.07, *p* = 0.002) decrease in spontaneous preterm labour following 1 January 2006 (Table 1). After adjustment for potential confounding factors (maternal age, sex of the infant, deprivation quintile, week of conception, number of previous spontaneous abortions, number of previous therapeutic abortions, and parity), the reductions in both measures were slightly greater in magnitude and remained statistically significant (Table 2). Inclusion of pre-eclampsia as a covariate attenuated the decrease in overall preterm deliveries slightly, but the step changes remained significant for both overall preterm delivery (−11.72, 95% CI −15.87, −7.35, *p*<0.001) and spontaneous preterm labour (−11.35, 95% CI −17.20, −5.09, *p* = 0.001).

**Table 1:** Univariate analysis of the step and slope per annum changes in pregnancy outcomes from 1 January 2006 referent to the underlying trend.

Outcome	Current Smokers				Never Smokers				Overall^a^			
	Step Change		Slope Change		Step Change		Slope Change		Step Change		Slope Change	
	Percent (95% CI)	*p*-Value	Percent (95% CI)	*p*-Value	Percent (95% CI)	*p*-Value	Percent (95% CI)	*p*-Value	Percent (95% CI)	*p*-Value	Percent (95% CI)	*p*-Value
**Small for gestational age**	−9.17 (−14.96, −2.98)	0.004	−1.84 (−5.08, 1.51)	0.279	−0.72 (−6.46, 5.37)	0.812	−2.35 (−5.18, 0.55)	0.112	−4.54 (−8.21, −0.73)	0.020	−2.68 (−4.54, −0.77)	0.006
**Very small for gestational age**	−15.05 (−24.89, −3.92)	0.009	0.21 (−5.88, 6.70)	0.948	0.04 (−12.39, 14.22)	0.996	−5.27 (−11.34, 1.21)	0.109	−7.82 (−14.95, −0.09)	0.048	−3.03 (−6.85, 0.94)	0.133
**Low birth weight**	−10.99 (−17.97, −3.41)	0.005	−2.29 (−6.28, 1.87)	0.276	−10.49 (−16.85, −3.51)	0.004	2.93 (−0.72, 6.71)	0.117	−9.53 (−13.82, −5.04)	<0.001	−1.08 (−3.42, 1.32)	0.376
**Preterm delivery**	−5.79 (−13.94, 3.14)	0.197	−0.14 (−4.61, 4.53)	0.951	−14.30 (−19.81, −8.40)	<0.001	5.10 (1.78, 8.53)	0.002	−11.07 (−15.15, −6.79)	<0.001	2.28 (−0.03, 4.66)	0.053
Mild	−7.79 (−17.15, 2.63)	0.138	2.47 (−2.89, 8.13)	0.373	−9.36 (−16.01, −2.19)	0.011	3.67 (−0.07, 7.56)	0.055	−8.22 (−13.11, −3.05)	0.002	2.60 (−0.09, 5.37)	0.058
Moderate	−9.71 (−28.76, 14.42)	0.398	−2.53 (−13.65, 10.03)	0.679	−24.36 (−37.49, −8.48)	0.004	7.38 (−2.09, 17.76)	0.131	−16.97 (−27.05, −5.49)	0.005	−0.17 (−6.39, 6.45)	0.958
Extreme	7.53 (−13.15, 33.13)	0.505	−8.60 (−18.16, 2.07)	0.110	−27.50 (−39.42, −13.22)	<0.001	10.24 (1.17, 20.13)	0.026	−16.60 (−25.92, −6.11)	0.003	2.40 (−3.37, 8.52)	0.422
**Spontaneous preterm labour**	−7.27 (−17.99, 4.84)	0.228	−1.80 (−7.81, 4.59)	0.572	−14.54 (−22.47, −5.80)	0.002	3.66 (−1.19, 8.75)	0.141	−10.26 (−16.07, −4.04)	0.002	0.20 (−3.06, 3.56)	0.908
Mild	9.73 (−21.78, 4.18)	0.161	−1.19 (−8.21, 6.37)	0.751	−11.14 (−20.37, −0.84)	0.035	2.25 (−3.13, 7.94)	0.420	−7.32 (−14.15, 0.05)	0.052	−0.49 (−4.19, 3.34)	0.798
Moderate	−4.98 (−31.57, 31.93)	0.760	−2.00 (−17.14, 15.90)	0.813	−25.69 (−45.04, 0.48)	0.054	8.29 (−6.56, 25.50)	0.290	−16.95 (−31.65, 0.93)	0.062	1.25 (−8.06, 11.51)	0.801
Extreme	3.79 (−23.85, 41.48)	0.814	−4.08 (−18.12, 12.38)	0.607	−23.39 (−41.97, 1.13)	0.060	8.30 (−5.34, 23.90)	0.246	−18.19 (−31.68, −2.04)	0.029	3.10 (−5.62, 12.63)	0.498

Data are step changes at 1 January 2006, and slope changes after 1 January 2006.

a Includes current, never, and former smokers and unknown smoking status.

**Table 2:** Multivariable analysis of the step and slope per annum changes in pregnancy outcomes from 1 January 2006 referent to the underlying trend.

Outcome	Current Smokers				Never Smokers				Overall^a^			
	Step Change		Slope Change		Step Change		Slope Change		Step Change		Slope Change	
	Percent (95% CI)	*p*-Value	Percent (95% CI)	*p*-Value	Percent (95% CI)	*p*-Value	Percent (95% CI)	*p*-Value	Percent (95% CI)	*p*-Value	Percent (95% CI)	*p*-Value
**Small for gestational age**	−7.95 (−13.90, −1.58)	0.015	−2.22 (−5.49, 1.17)	0.198	−2.45 (−8.21, 3.66)	0.423	−1.92 (−4.80, 1.06)	0.204	−4.52 (−8.28, −0.60)	0.024	−1.54 (−3.47, 0.44)	0.126
**Very small for gestational age**	−15.09 (−25.10, −3.74)	0.011	0.90 (−5.34, 7.54)	0.784	−1.69 (−14.08, 12.48)	0.804	−5.29 (−11.43, 1.27)	0.112	−7.95 (−15.19, −0.08)	0.048	−1.23 (−5.17, 2.88)	0.553
**Low birth weight**	−10.59 (−17.76, −2.81)	0.009	−1.80 (−5.89, 2.47)	0.402	−11.26 (−17.80, −4.21)	0.002	4.47 (0.67, 8.41)	0.021	−9.85 (−14.24, −5.23)	<0.001	0.89 (−1.56, 3.41)	0.478
**Preterm delivery**	−5.51 (−13.84, 3.63)	0.229	0.51 (−4.06, 5.30)	0.830	−15.44 (−21.02, −9.47)	<0.001	6.90 (3.44, 10.48)	<0.001	−11.72 (−15.87, −7.35)	<0.001	3.83 (1.42, 6.30)	0.002
Mild	−6.94 (−16.55, 3.76)	0.195	2.98 (−2.49, 8.76)	0.292	−10.90 (−17.56, −3.69)	0.004	5.32 (1.43, 9.35)	0.007	−8.78 (−13.74, −3.54)	0.001	4.06 (1.27, 6.92)	0.004
Moderate	−9.94 (−29.13, 14.44)	0.392	−2.51 (−13.78, 10.23)	0.685	−24.15 (−37.59, −7.81)	0.005	8.82 (−0.98, 19.60)	0.079	−17.03 (−27.30, −5.32)	0.006	0.98 (−5.42, 7.82)	0.770
Extreme	5.59 (−15.17, 31.42)	0.626	−7.01 (−16.93, 4.10)	0.207	−27.19 (−39.42, −12.49)	0.001	12.60 (3.15, 22.92)	0.008	−17.41 (−26.86, −6.73)	0.002	4.27 (−1.73, 10.65)	0.167
**Spontaneous preterm labour**	−8.85 (−19.56, 3.28)	0.146	−0.62 (−6.78, 5.95)	0.849	−15.56 (−23.53, −6.76)	0.001	4.15 (−0.81, 9.35)	0.102	−11.35 (−17.20, −5.09)	0.001	1.16 (−2.19, 4.62)	0.502
Mild	−10.36 (−22.48, 3.67)	0.141	−0.26 (−7.43, 7.47)	0.946	−13.11 (−22.26, −2.88)	0.013	3.20 (−2.32, 9.02)	0.262	−8.56 (−15.40, −1.17)	0.024	0.56 (−3.23, 4.49)	0.777
Moderate	−5.17 (−32.04, 32.33)	0.755	−1.78 (−17.17, 16.47)	0.836	−25.07 (−44.91, 1.92)	0.066	6.84 (−8.10, 24.22)	0.389	−18.09 (−32.88, −0.05)	0.049	2.23 (−7.37, 12.82)	0.661
Extreme	−3.96 (−30.18, 32.09)	0.804	−1.01 (−15.83, 16.42)	0.902	−19.78 (−39.68, 6.69)	0.130	7.14 (−6.61, 22.91)	0.325	−18.24 (−32.04, −1.63)	0.033	3.54 (−5.43, 13.36)	0.452

Data are step changes at 1 January 2006, and slope changes after 1 January 2006. Data adjusted for maternal age, infant sex, deprivation quintile, week of conception, number of previous spontaneous abortions, number of previous therapeutic abortions, and parity.

a Includes current, never, and former smokers and unknown smoking status.

**Figure 2:**
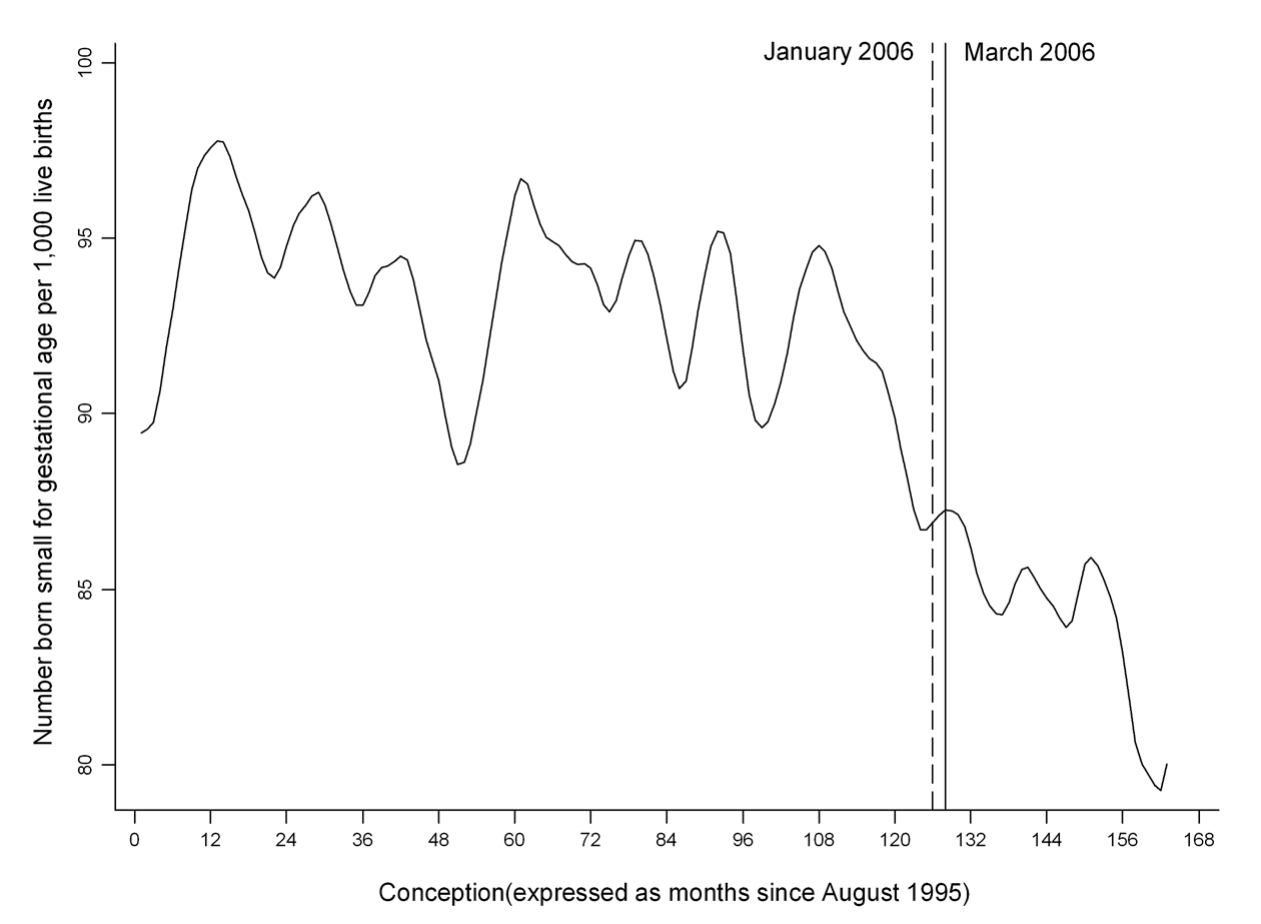
Time trend in the number of infants delivered small for gestational age per 1,000 live births. Time trend smoothed using the Stata loess smoother with bandwidth = 0.1.

**Figure 3:**
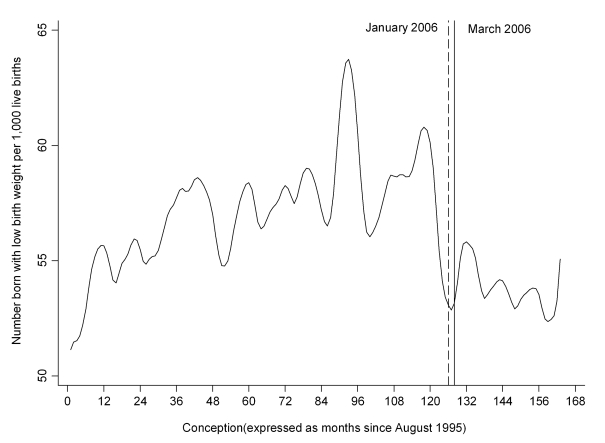
Time trend in the number of infants delivered with low birth weight per 1,000 live births. Time trend smoothed using the Stata loess smoother with bandwidth = 0.1.

Figure 2 depicts the crude proportion of infants delivered small for gestational age by month of conception. Overall, there was a 4.54% (95% CI 0.73, 8.21, *p* = 0.020) decrease in the number of infants born small for gestational age and a 7.82% (95% CI 0.09, 14.95, *p* = 0.048) decrease in those born very small for gestation age at the breakpoint 1 January 2006 (Table 1). The results were similar after adjustment for potential confounding factors (Table 2). Addition of pre-eclampsia to the multivariable model had little effect, with the step changes remaining statistically significant for both small (−4.52%, 95% CI −8.28, −0.60, *p* = 0.024) and very small (−7.95%, 95% CI −15.19, −0.08, *p* = 0.048) infants. Low birth weight also demonstrated a significant step change on 1 January 2006 (Tables 1 and 2; Figure 3).

Imputation of missing smoking status had little impact on the multivariable results. Following 1 January 2006, there remained significant step decreases in small for gestational age (−4.30, 95% CI −8.00, −0.45, *p* = 0.029), preterm delivery (−12.43, 95% CI −16.53, −8.12, *p*<0.001), and spontaneous preterm labour (−11.71, 95% CI −17.53, −5.47, *p* = 0.001).

## Discussion

In this study, introduction of national, comprehensive smoke-free legislation was associated with significant reductions in preterm delivery and small for gestational age. Reductions in adverse pregnancy outcomes were observed among both mothers who smoked and mothers who had never smoked. These results are plausible. Active maternal smoking has detrimental effects on placental architecture, placental function, and early and late foetal growth [17]–[21], predisposing to a range of pregnancy complications, including moderate and extreme preterm birth, intrauterine growth restriction, and low birth weight [3]–[7]. Maternal exposure to ETS is also harmful. A recent meta-analysis of 78 studies demonstrated increased risk of low birth weight and intra-uterine growth restriction [7]. A causal relationship is supported by the results of a recent study in which infants born to mothers who were randomized to an intervention that reduced ETS exposure had significantly lower risk of very low birth weight (<1,500 g) (odds ratio 0.11, 95% CI 0.01, 0.86) and very preterm delivery (<34 wk) (odds ratio 0.22, 95% CI 0.07, 0.68) [2].

The effects on pregnancy complications preceded implementation of the legislation by 3 mo. This is consistent with a previous study in which we demonstrated that smokers anticipated the legislation, resulting in a significant peak in nicotine replacement therapy prescriptions in January 2006 [11]. The initial fall in smoking prevalence that occurred as a result has not been maintained [11], but there has been a reduction in the amount smoked by those who continued or resumed active smoking following the legislation [10]. It is difficult to extrapolate changes in smoking behaviour among the general population to pregnant women, but the reduction in smoking prevalence that we demonstrated among pregnant women is plausible. Irrespective of legislation, many women quit smoking when pregnant because of concerns regarding their infant's health [22], and there has been increased awareness of the need to protect children following the Scottish legislation, resulting in an increase in voluntary home restrictions [9] and reduced ETS exposure among children as well as adults [9],[10]. Following the Italian smoke-free legislation, smoking prevalence among pregnant women decreased at conception and during the first trimester, but this decrease did not reach statistical significance [23], and following the Irish legislation, smoking prevalence for pregnant women decreased by 12% [24].

One previous study has examined the effect of smoke-free legislation on pregnancy complications. A single-centre retrospective study conducted in Ireland compared pre- and post-legislation pregnancy complications among 15,241 women [15]. The investigators were not able to take account of underlying trends. This is important since both preterm deliveries and birth weight have been increasing over a number of years, largely because of increases in the frequency of elective preterm delivery and maternal body mass index, respectively. The investigators in the Ireland study demonstrated a reduction in preterm deliveries among active smokers but were unable to differentiate between spontaneous preterm labour and elective preterm deliveries, which have different aetiologies. Smoking is associated with an increased risk of intra-uterine infections and a systemic inflammatory response, both of which can induce labour [24]. Therefore, smoking is strongly associated with spontaneous preterm labour [25]. In contrast, smoking has a protective effect against pre-eclampsia, which is a common indication for elective preterm delivery. The reduction we demonstrated in preterm birth was only slightly attenuated following adjustment for pre-eclampsia, and remained statistically significant. This suggests that any detrimental effect the legislation may have had on pre-eclampsia has been more than offset by beneficial effects on other conditions. The study in Ireland also demonstrated a significant increase in low birth weight following the implementation of smoke-free legislation. However, the study reported only absolute birth weight, which is determined, in large part, by gestation at delivery. This contrasts with our findings of a reduction in both small and very small for gestational age, as well as in absolute low birth weight.

The main strengths of our study are its large scale and that it covered all pregnancies in Scotland, thereby avoiding selection bias. We were able to account for underlying trends in pregnancy complications prior to implementation of the legislation and could examine spontaneous preterm deliveries as well as all preterm deliveries. We used routinely collected data: the data are subjected to regular quality assurance checks, and their quality is high. In contrast with the Irish study, we were able to take account of underlying trends prior to implementation of the legislation. In addition to overall changes, we were able to report changes among the sub-groups of current and never smokers. We did not undertake sub-group analysis on former smokers. If implementation of the legislation led to an increase in smoking cessation, the sub-group of former smokers will contain a higher percentage of individuals who only recently stopped smoking in the post-legislation period. The risks associated with active smoking decline over time following cessation. Therefore, a comparison of pre-legislation former smokers and post-legislation former smokers would be subject to bias.

The main limitation of our study was that smoking status was based on self-classification. Pregnant women have been shown to underestimate their smoking prevalence by up to 25% [26]. More importantly, it is plausible that women felt greater pressure to conceal active smoking following implementation of the legislation, leading to systematic error. Individuals who smoke but do not identify themselves as smokers tend to classify themselves as former, rather than never, smokers [27]. Therefore, an increase in smokers classifying themselves otherwise post-legislation could introduce systematic error into the analysis of self-reported current smokers. However, it would not affect the overall results, which relate to all deliveries irrespective of maternal smoking status.

Birth weight centiles were derived from data across the whole study period. Inclusion of post-legislation data may have resulted in an underestimate of the true impact of the legislation. We were unable to examine pre-eclampsia as a secondary outcome because it is known to be under-recorded. We are not aware of any changes in obstetric practice that coincided with implementation of the legislation and may have impacted on the study outcomes. We did not have access to reliable data on maternal obesity and height. However, these factors are unlikely to have introduced systematic error. Maternal obesity has increased over the whole period, and whilst maternal obesity increases the risk of elective preterm delivery, it is protective against spontaneous preterm delivery [28].

Consistent with many countries, Scotland has experienced an increase in both spontaneous and iatrogenic preterm birth rates. Survival among infants delivered preterm has improved. However, these infants remain at increased risk of long-term neurodevelopmental sequelae and generate substantial healthcare and societal costs. Any intervention that can reduce the risk of preterm delivery has the potential to produce important public health benefits. The results of our study add to the growing evidence of the wide-ranging health benefits of smoke-free legislation and lend support to the adoption of such legislation in countries where it does not currently exist.

## Abbreviations

ETS: environmental tobacco smoke
SMR2: Scottish Morbidity Record
